# The Potential of Post-Mortem Carcass Assessments in Reflecting the Welfare of Beef and Dairy Cattle

**DOI:** 10.3390/ani9110959

**Published:** 2019-11-13

**Authors:** Melody Knock, Grace A. Carroll

**Affiliations:** 1 Animal Behaviour and Welfare Research Group, Department of Animal and Land Sciences, Hartpury University, Gloucester GL19 3BE, UK; melodyknock1995@hotmail.co.uk; 2 Animal Behaviour Centre, School of Psychology, Queen’s University Belfast, Belfast, BT7 1NN, UK

**Keywords:** abattoir, animal welfare, ante-mortem, beef, dairy, post-mortem

## Abstract

**Simple Summary:**

Information recorded at abattoirs may be useful for informing farmers of the health and welfare of their herds. This information can also be used to identify where and when welfare problems occur in the production chain. This study aimed to determine whether welfare issues in live beef and dairy cattle were reflected in post-mortem measures. A number of ante-mortem issues, including lameness and poor body condition, were reflected in post-mortem assessments. However, these varied according to the breed of cattle. While more research is needed, the results suggest that post-mortem assessments in cattle may be useful in reflecting the welfare of the live animal. Meat inspection currently focuses on human health and food safety. Adapting meat inspection processes to allow for greater consideration of animal welfare would be beneficial given the increased public interest in the welfare of food animals.

**Abstract:**

There is increasing interest in utilizing meat inspection data to help inform farmers of the health and welfare of their herds. The aim of this study was to determine whether ante-mortem measures of welfare in beef and dairy cattle (N = 305) were associated with post-mortem measures at a United Kingdom (UK) abattoir. Multiple regression analysis was used to determine the ability of ante-mortem measures of lameness, cleanliness, skin lesions, hair loss and body condition in predicting hot carcass weight and the frequency of carcass bruising. For beef cattle, lameness score (*p* = 0.04), cleanliness score (*p* = 0.02) and age (*p* < 0.001), were predictors of carcass bruise score while lameness score (*p* = 0.03), body condition (*p* = 0.01) and sex (*p* < 0.001) were predictors of hot carcass weight. For dairy cattle, sex (*p* < 0.001) and slaughter day (*p* < 0.001) were predictors of carcass bruise score while skin lesion score (*p* = 0.01), body condition (*p* < 0.001), age (*p* < 0.001), slaughter day (*p* < 0.001) and number of moves (*p* = 0.01) were predictors of hot carcass weight. These results suggest that recording carcass weight and carcass bruising at meat inspection may have potential as a general indicator of health and welfare status in cattle. However, animal characteristics and variables, such as slaughter day and abattoir staffing, should be taken into account when interpreting the results.

## 1. Introduction

Animal production is constantly evolving to accommodate the rapidly increasing demand for animal-based products in the most efficient and environmentally sustainable way possible [1]. The pressure to reduce labour costs has resulted in greater animal to human ratios on farms which decreases time allocations for each animal, increasing the likelihood of inadequate inspections [2]. In addition, animals encounter several risks to their welfare during the marketing process associated with overcrowding of lorries, mixing of unfamiliar animals, rough handling and goad use [3,4]. These problems can be exacerbated by commercial pressures such as maintaining processing speed [5].

It is in the interest of producers and processors to be concerned with the welfare of their animals as poor welfare impacts productivity and results in economic losses [6,7]. For example, processors must trim bruised meat from the carcass, incurring losses for the processor and/or farmer as a result [6]. Consequently, identifying the source of this damage is important. Maintaining high levels of welfare also avoid violation of animal welfare legislation, which can result in penalties [8]. Meat inspection was originally implemented to identify meat unsafe for human consumption as a result of the transmission of infectious agents in contaminated meat [9]. It was later recognised as a sufficient way of monitoring diseases relating to animal health and welfare [10]. There is now increasing interest in utilizing meat inspection data further to help inform farmers of the health and welfare of their herd to improve management of farmed species. In addition, monitoring of patterns within meat inspection data is being increasingly used for early detection of diseases and to identify animal welfare issues arising during the transportation and lairage stages of production [3,11]. Integrating welfare surveillance into standard meat inspection is an inexpensive way to monitor animal health as no additional costs may be added [12]. Collecting animal welfare-related data within the abattoir also has the potential to reduce reliance on on-farm assessments which may be logistically difficult. For example, dairy cattle can be overstocked [13] and outdoor cattle can control their proximity to humans [14]. Both of these factors may increase the difficulty of carrying out welfare assessments on-farm. The results of the inspection can be utilized by processors to make improvements and can also be fed back to farmers in a constructive manner via the farms veterinarian, who, of authoritative standing, may influence the farmer to improve standards [15].

Existing meat inspection measures are not necessarily relevant to animal welfare [16]. A number of studies have explored the potential of integrating welfare measures into routine abattoir processes in pigs [17,18,19] and the European Union (EU) Broiler Directive explicitly mentions animal-based indicators, such as footpad dermatitis, that should be assessed at the abattoir as potential indicators of poor welfare conditions [20]. However, less research has been carried out to explore the potential of different abattoir-based welfare assessment methods in cattle. In contrast to pigs, cattle are skinned as part of the slaughter process [21]. Consequently, visual assessment of the carcass may be necessary at multiple points—pre- and post-skinning—as it is unknown whether carcass bruising in cattle reflects visual injury or damage that can be seen ante-mortem. ‘Iceberg indicators’ are welfare measures thought to provide an overall assessment of welfare, being potentially indicative of further problems [22]. It is important to ensure that any ante or post-mortem welfare measures selected for inclusion in a routine assessment of cattle welfare are feasible to assess in a fast-paced abattoir environment [23]. Therefore, reducing the number of measures required for such assessments should be a key aim. If post-mortem indicators of carcass bruising and carcass weight were to reflect issues present ante-mortem, this would simplify abattoir-based welfare assessments by reducing the number of measures that it is necessary to assess on the live animal.

The aim of this study was to determine if ante-mortem measures in cattle were associated with post-mortem measures and, if so, to determine animal-based measures to be included in routine welfare assessments.

## 2. Materials and Methods

Three-hundred and five beef (n = 123) and dairy (n = 182) cattle from an abattoir in south-west England were assessed. In total, 16.4% of cattle were bulls (beef = 13.1%, dairy = 18.7%), 17.8% were steers (beef = 18%, dairy = 17.6%), 61.8% were cows (beef = 62.3%, dairy = 61.5%) and 3.9% were heifers (beef = 6.6%, dairy = 2.2%). This abattoir processes beef cattle, breeding stock and cull cows. Consequently, ages of animals at slaughter ranged from 1 year and 1 month (417 days), to 18 years and 7 months (6818 days) with a mean age of 5 years and 3 months (1918 days ± 1311). A total of 68.3% of beef cattle and 64.3% of dairy cattle were cows. Data was collected over a period of five days between January and February 2017.

### 2.1. Abattoir Handling and Slaughter Practices

The British Cattle Movement Service (BCMS) is responsible for maintaining information regarding all bovine animals in the United Kingdom (UK) [24]. All bovines are assigned a unique number at birth, which is displayed via an ear tag. This number is recorded on the animals’ passport, which is held by the current keeper of the animal [25]. The passport and unique number remain with the animal throughout its entire life and is recorded at the abattoir at the time of death. The passport contains details of the animal including its birth holding, ear-tag number, date of birth, breed, sex and mothers’ ear tag number [26]. When the animal is moved from its original holding, the movement date and new holding number must be recorded each time.

Prior to unloading, passports for each individual animal were checked. Cattle were then unloaded from the transportation lorries and held in lairage pens. A rectangular plastic paddle was used to drive the cattle to their assigned pen. Within the lairage, each animal was assigned a slaughter number, attached to the ear, which was used as a means of identification throughout the entire slaughter process. Once a batch of cattle were required for slaughter, cattle were moved by an operator from the lairage pen to the race. The race leads from the holding pen via a straight corridor that guides cattle around a smooth corner to the stun box. The stunning box is separated from the corridor by a non-return gate that restricts the entrance to the next animal in line. Cattle were sorted into single file into the race by two operators who checked ear tags and animals were then driven into the stunning box. One operator used an electric goad, when deemed necessary, to encourage cattle into the stunning box where a captive bolt pistol was used to stun cattle prior to slaughter. After cattle were stunned, they were dropped from the stunning box into the slaughter hall below and hung by their hind leg in readiness for exsanguination. After exsanguination, the cattle were processed through a variety of stations that removed certain body extremities such as the ears and lower leg.

### 2.2. Inter-rater Reliability

Two observers were required on each data collection occasion. Inter-rater reliability scoring was carried out before data collection commenced. The scoring systems used in the study were first viewed and discussed by both observers in order to gain a consensus as to what score should be assigned in various circumstances. Ten animals were used in the training session whereby both observers scored the animals. Any discrepancies between assigned scores were discussed. Subsequently, 30 animals were randomly assigned to be independently and blindly scored for each animal-based measure by both observers. Any discrepancies were discussed and scoring repeated over five sessions [27]. The interclass correlation coefficient test was used to determine the overall level of reliability between raters. A value of 0.740 was derived, which is classified as strong reliability between raters [28].

### 2.3. Data Collection

Data collection began at various times on each data collection day, dependent on the arrival of animals, with an earliest starting time of 07:30 and a latest starting time of 16:30. One researcher was located within the lairage while a second was located within the slaughter hall. Observers alternated between these positions on a daily basis.

In order to assess lameness, Observer 1 was positioned in a safe location, behind a barrier, which was parallel to a concrete-floored unloading area. Cattle were scored for lameness as they were unloaded from the lorries and moved towards the lairage pens. Each animal was then scored for cleanliness, lesions and hair loss and body condition, from an elevated vantage point, subsequent to being moved from the lairage pen to the race. The researchers had sufficient time needed to record each of the animal-based measures. However, as the animals could not be assessed at close proximity it is possible that some signs of injury could have gone unnoticed. Handheld transceivers were used as a means of communication between researchers, in order to ensure that the animals scored within the lairage were also being scored for bruising in the slaughter hall. Within the slaughter hall, Observer 2 was positioned directly opposite the deskinning area. There was a slow line speed with 250 to 300 animals slaughter per day on an ad hoc basis.

### 2.4. Welfare Assessment Measures

#### 2.4.1. Ante-mortem Indicators

Lameness Scoring

A mobility scoring system developed by the Agriculture and Horticulture Development Board (AHDB) [29] was used on both beef and dairy cattle. Scores were; (0) good mobility, even weight bearing on all four limbs; (1) imperfect mobility, uneven steps; (2) impaired mobility, uneven weight bearing; (3) severely impaired mobility, unable to walk at the same pace as the rest of the herd

Cleanliness Scoring

Cleanliness was scored using a system developed by AHDB [30] on a scale of 0 to 2 and were; (0) clean, no dirt only minor splashing present; (1) dirty, an area of dirtiness at least 10 × 15 cm; (2) very dirty, plaques of dirt at least 40 cm in any dimension.

Lesion and Hair Loss Scoring

Lesions were defined as any abnormality to the skin as a result of injury or disease, which included sores, cuts, scratches and skin infections [31]. Lesions and hair loss were scored using a system developed by the AHDB [32]. The body, neck, tail and legs were assigned a score of either (0) no lesions or hair loss; (1) one or more hairless patches larger than 2 cm in diameter; or (2) one or more lesions larger than 2 cm in diameter. Lesions occurring anywhere other than the ‘neck,’ ‘tail’ and ‘legs’ regions were classified under the heading of ‘body.’ If there was more than one lesion in one of the specified body regions, the maximum severity was recorded.

Body Condition Scoring (BCS)

Due to differences in body conformation between beef and dairy cattle, two body condition scoring (BCS) systems were used (see Department for Environment, Food and Rural Affairs (DEFRA) for beef cattle [33] and for dairy cows [34]. Both scoring systems had similar technical descriptions. However, a visual scoring system was also utilised and allowed the conformation differences between beef and dairy breeds to be accounted for. Scores ranged from (1) Poor, (2) Moderate, (3) Good, (4) Fat to (5) Grossly fat. Body condition score was condensed into a binary variable for the regression analysis; score 1 and 2 were combined to form the category ‘Underweight,’ scores 3 to 5 were combined to form the category ‘Not underweight.’

#### 2.4.2. Post-mortem Indicators

Bruise Scoring

Bruising is defined as the rupture of blood vessels that results in haemorrhage of tissue, causing accumulation of blood that discolours beneath the surface of the skin [35,36]. The size, shape and colour of bruising were assessed using Strappini et al.’s [37] scoring system. See Strappini et al. [37] for figures outlining the location and shape of bruises. Briefly; seven anatomical sites were used to record bruise location—(1) Butt, (2) Rump-loin, (3) Rib, (4) Forequarter, (5) Back, (6) Pin and (7) Hip [37]. The size of the bruise at each of the seven anatomical sites was scored based on the diameter or longest length of bruise and assigned a score from 1 to 4—(1) 0 cm–2 cm, (2) 2 cm–8 cm, (3) 9 cm–16 cm, (4) more than 16 cm [37]. If more than one bruise was present on a specific area, the maximum severity of the size were recorded. Bruise size was determined by holding up a clipboard with an integrated ruler to each bruise. The shape of bruise was recorded as either circular; a bruise shaped like or nearly like a circle, linear; a non-circular bruise with one dimension (length) longer than the other (width), tramline; two parallel linear bruises separated by a paler undamaged area, mottled; the bruised area appears spotted or blotched or irregular; a bruise without clear dimensions and with uneven margins [37]. If there was more than one bruise shape present in a specific body region, that bruising was recorded as ‘multiple.’ The colour of the bruise was classified into one of three colours; (1) Red, (2) Purple or (3) Yellow [37], which indicate a fresh, old or very old bruise, respectively.

#### 2.4.3. Other Measures

Breed codes for each animal were obtained from abattoir records. Holstein Friesian cattle and associated variants (‘Holstein,’ ‘Holstein Friesian cross’ and ‘Holstein cross’) were recorded as ‘dairy’ and any other breed was reported as ‘beef.’ Official cattle breed codes were checked to ensure correct classification as a beef or dairy breed [38]. Sex, age, farm of origin and number of moves during the lifetime were recorded in the lairage using the information available on each passport. Hot carcass weights and condemnation information were obtained for all animals from abattoir records subsequent to the completion of data collection. In line with the mandatory Beef Carcase Classification Scheme [38], carcasses were graded by trained technicians for conformation (from 1 [poor] to 6 [superior]) and fat coverage (from 1 [low] to 5 [very high]). These data were obtained from abattoir records.

### 2.5. Ethics

The research proposal was approved by Hartpury University ethics committee (ETHICS2015-36). All data was collected using non-invasive methods.

### 2.6. Statistical Analysis

Descriptive statistics were used to determine the frequency of bruises, lameness, cleanliness, lesions, hair loss and body condition scores. Multiple regression analysis was used to determine the ability of ante-mortem measures (lameness, cleanliness, lesions and hairless patches and body condition), age in days, sex (male or female), slaughter day and number of moves in predicting the frequency of carcass bruising and hot carcass weight. Separate analyses were carried out for beef and dairy cattle. Non-significant variables were removed using Backward selection. Ante-mortem animal-based measures were condensed into categorical binary dummy variables for analysis (0 = absent, 1 = present, for ante-mortem measures and 0 = one move, 1 = more than one move, for number of moves). Spearman’s’ correlations were used to assess associations between body condition score (1–5) and conformation and fat coverage grades for beef and dairy cattle. All statistical analyses were carried out using IBM SPSS, version 24.

## 3. Results

**Figure 1:**
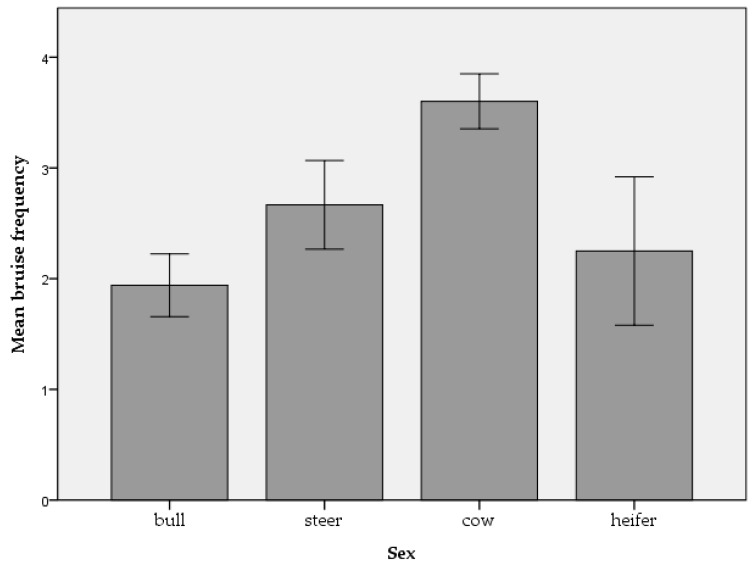
Mean bruise frequency for bulls, steers, cows and heifers.

**Figure 2:**
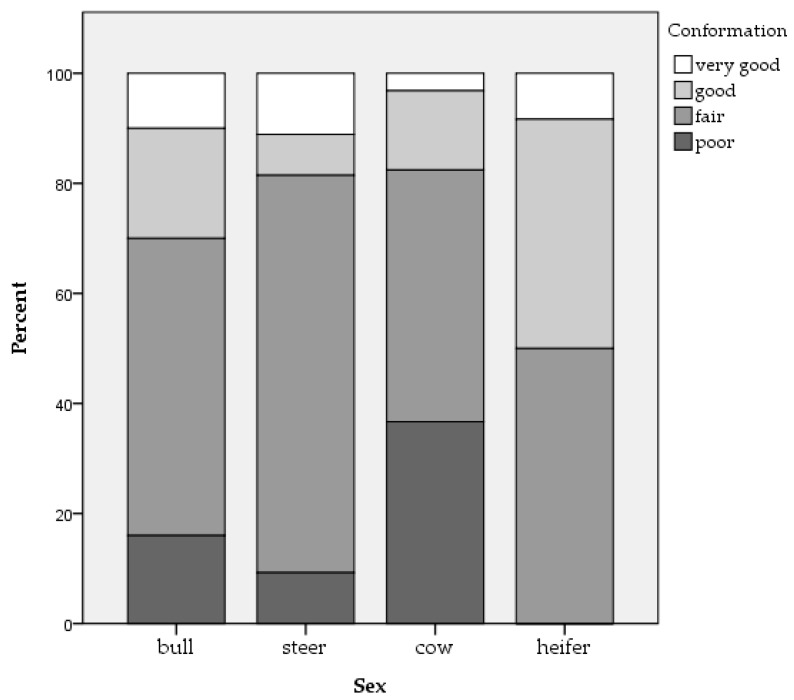
Percentage of bulls, steers, cows and heifers within each of the conformation grades.

**Figure 3:**
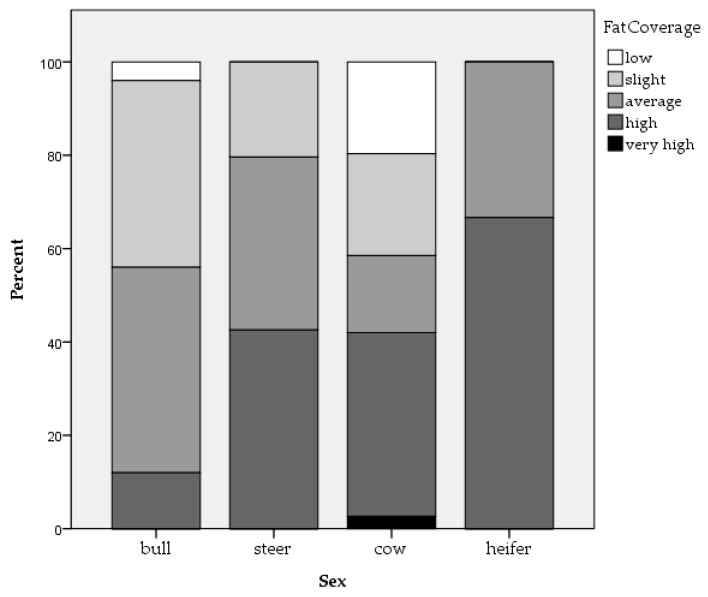
Percentage of bulls, steers, cows and heifers within each of the fat coverage grades.

Three hundred and five beef (n = 123) and dairy (n = 182) cattle from 40 farms were assessed, with an average of eight cattle per batch. Two whole carcass condemnations were reported, both as a result of the detection of oedema. Of the 305 cattle, it was possible to obtain movement data for 303 animals. In total, 57.1% of cattle were moved once, 23.8% were moved twice, 12.2% were moved three times, 4.6% were moved four times and 2.3% of cattle were moved between five and eight times. Mean slaughter age in days was 2064.93 (±1254.38) for beef cattle and 1818.72 (±1382.130) for dairy cattle. The percentage of beef and dairy cattle respectively with each ante-mortem welfare issue is outlined in Table 1. Mean bruise frequency and carcass grading measures of conformation and fat coverage can be seen in Figure 1, Figure 2 and Figure 3 for each category of cattle (bull, steer, cow and heifer). Note that only 3.9% of the assessed cattle were heifers. No cattle were classified as having ‘excellent’ or ‘superior’ conformation. Therefore, these categories are not included in Figure 2. There was a strong association between body condition score and conformation grade in beef cattle (r = 0.583, *p* < 0.001) and dairy cattle (r = 0.560, *p* < 0.001). There was a moderate association between body condition score and fat coverage grade in dairy cattle (r = 0.493, *p* < 0.001) and a weak association in beef cattle (r = 0.199, *p* = 0.028).

### 3.1. Bruise Characteristics in Beef and Dairy Cattle

Bruise characteristics in beef and dairy cattle with regard to shape, location and colour are outlined in Table 2 and Table 3.

### 3.2. Associations between Ante-Mortem and Post-Mortem Welfare Indicators—Beef Cattle

#### 3.2.1. Carcass Bruising

In beef cattle, 20.2% of the variation in carcass bruise frequency could be explained by the model (F = 10.81, *p* < 0.001). Subsequent to backward selection, there was a significant effect of age (*p* < 0.001, B (SE) = 0.001 (0.00), β = 0.39), lameness (*p* = 0.042, B (SE) = 1.39 (0.67), β = 0.17) and cleanliness (*p* = 0.016, B (SE) = 0.91 (0.37), β = 0.21) on carcass bruising with increased bruise frequency being associated with older slaughter age and being lame and dirty ante-mortem.

#### 3.2.2. Hot Carcass Weight

In beef cattle, 16.5% of the variation in hot carcass weight could be explained by the model (F = 8.66, *p* < 0.001). Subsequent to backward selection, there was a significant effect of sex (*p* = 0.001, B (SE) = 56.59 (16.49), β = 0.30), lameness (*p* = 0.032, B (SE) = 74.20 (34.16), β = 0.19) and body condition (*p* = 0.008, B (SE) = 62.24 (22.95), β = 0.24) on hot carcass weight. Specifically, lower hot carcass weight was associated with being female, being lame and having a poor body condition.

### 3.3. Associations between Ante-Mortem and Post-Mortem Welfare Indicators—Dairy Cattle

#### 3.3.1. Carcass Bruising

In dairy cattle, 21.8% of the variation in carcass bruise frequency could be explained by the model (F = 25.62, *p* < 0.001). Subsequent to backward selection, there was a significant effect of slaughter day (*p* < 0.001, B (SE) = −1.09 (0.22), β = −0.33) and sex (*p* < 0.001, B (SE) = −1.03 (0.23), β = −0.30) on carcass bruising with increased bruising in females.

#### 3.3.2. Hot Carcass Weight

In dairy cattle, 33.2% of the variation in hot carcass weight could be explained by the model (F = 18.63, *p* < 0.001). Subsequent to backward selection, there was a significant effect of age (*p* < 0.001, B (SE) = 0.015 (0.003), β = 0.28), slaughter day (*p* < 0.001, B (SE) = 41.98 (8.30), β = 0.34), number of moves (*p* = 0.013, B (SE) = −20.53 (8.44), β = −0.16), lesions (*p* = 0.010, B (SE) = −29.30 (16.06), β = 0.12) and body condition (*p* < 0.001, B (SE) = 59.04 (8.37), β = 0.46) on hot carcass weight. Specifically, lower hot carcass weight was associated with younger slaughter age, more moves and having lesions and poor body condition ante-mortem.

See Table 4 for a summary of the significant predictors of carcass bruising and hot carcass weight for beef and dairy cattle.

## 4. Discussion

The use of abattoir-based welfare assessments as a health and welfare monitoring tool is increasingly being explored across a number of farmed species [12,17,19,39]. Live cattle can demonstrate dangerous behaviours such as kicking and struggling prior to slaughter [40] and post-mortem assessments could reduce the need to carry out potentially dangerous assessments on farms and at ante-mortem inspection.

### 4.1. Carcass Bruising

The current study assessed the ability of ante-mortem measures to predict carcass bruise frequency and hot carcass weight in beef and dairy cattle. A very high incidence of bruising was observed in the current study for both beef and dairy cattle. Previous studies have varied considerably in reported bruise prevalence. For example, Strappini et al. [35] reported a bruise prevalence of 8.58% and 20.76% in two different abattoirs. However, these figures were taken from routine meat inspection records. Meat Inspectors at pork processing plants often report lower prevalence of health and welfare issues than are reported elsewhere (e.g. [41]) and this may also be the case at beef abattoirs and would explain the large differences seen in reported prevalence. Similar to the current study, a number of other researchers reported that 90% or more of cattle assessed post-mortem had bruises [42,43,44]. In these studies, assessments were carried out by the researchers rather than meat inspectors and may be more indicative of actual bruise prevalence within the observed animals. Due to the high prevalence of bruises noted in the current study, it seems important that bruise scoring be included in any welfare-centred assessment scheme for cattle. However, given the high prevalence, it may be necessary to establish a baseline level of bruising [4]. The proportion of carcass bruising that can be attributed to any specific production stage is unknown. Strappini et al. [45] found that 67.3% of bruises in dairy cattle assessed post-mortem could be traced back to events encountered at the abattoir, with 38.5% of bruising sustained in the hour prior to slaughter. Dairy cattle, in particular, may be susceptible to sustaining injury during the marketing process as they may have pre-existing issues prior to transportation that are then exacerbated [46]. It is possible that some of the observed bruising was sustained on the farm of origin. However, in the current study, the majority of bruises were red; indicating that they were quite recently sustained [37]. In addition, despite the differences in environmental conditions and husbandry practices that beef and dairy cattle are subject to on-farm, bruise characteristics for both animal categories followed a similar pattern (see Table 2 and Table 3). This could suggest that bruising was sustained at the abattoir. The general order in which bruises change colour is widely agreed on across species [47,48,49]. However, using colour as an indicator of bruise age is unreliable. For example, bruising over a bony prominence will appear more quickly than a deep bruise within the muscle tissue that may not appear immediately [50]. Indeed, while difficult to differentiate, bright red and dark red bruises may differ in age with bright red bruises indicate damage that is 0 to 10 h old, and dark red bruises reflect those that are between 11 to 24 h old [49,51]. In the current study, a distinction was not made between bright and darker red bruising and making such a distinction may not be feasible at a commercial level.

### 4.2. Associations between Ante-Mortem and Post-Mortem Measures

Lameness and cleanliness were associated with increased bruise frequency in beef cattle but not dairy cattle. It is possible that animals subject to adverse conditions during marketing may have been at risk of sustaining bruising in addition to lameness and reduced cleanliness. For example, bad driving habits and poorly maintained lorries could result in increased falls and trips and this could lead to the animals sustaining injuries. In addition, stressful events such as transportation may increase the levels of defecation, which may soil the coat of the animals [52]. It is somewhat surprising that hot carcass weight was related to lameness in beef cattle but not in dairy cattle. Lameness is painful and reduces the ability of the animal to express normal behaviour such as competing for food [53,54]. Previous research suggests that lameness in dairy cattle is associated with both poor body condition and low body weight, independent of body condition [55,56,57]. However, Westin et al. [55] found that an inability to fit the stall width was the biggest animal-based risk factor for lameness in dairy cattle. Therefore, it is possible that dairy cattle of varying sizes may be subject to different risks for lameness and this could partially explain the findings of the current study. However, this is speculative and further research is needed. Skin lesions were a predictor for lower hot carcass weight in dairy cattle. Skin lesions have been linked to environmental and husbandry factors on-farm. Furthermore, in dairy cattle, lesions can be seen in exposed areas such as the tarsal joints [58]. Inorganic bedding is favoured by some dairy farmers as it removes the risk of bacterial growth associated with organic bedding materials such as straw [59]. However, bedding material such as rubber mats or concrete are not malleable to the shape of the cows body and may be less comfortable than straw or more supple inorganic materials such as sand [60]. Difficulty in lying down due to factors such as lameness or a narrow lying space may result in rubbing or bumping against the stall, resulting in damage to exposed areas [60]. The association between skin lesions and lower carcass weight could reflect an increased risk of such skin damage in thinner dairy cattle.

Carcass bruising was not associated with body condition. This finding is in contrast to that of Sánchez-Hidalgo et al. [56] who found an association between carcass bruising in cull cows and poor body condition ante-mortem. Sánchez-Hidalgo et al. [56] created a bruise score that combined bruise frequency, size and depth while bruise frequency alone was used in the current study. Future research should consider the associations between different bruise characteristics and ante-mortem welfare indicators. Lower hot carcass weight was associated with poor body condition for both beef and dairy cattle. It is logical that animals with poor body condition will have a lower carcass weight. Poor body condition can be the result of disease such as a displaced abomasum or can signal poor nutritional management [61,62]. Consequently, poor body condition can be said to be an indicator of long-term reduced health and welfare [39]. This finding, to some extent, validates the accuracy of the body condition scoring systems used within the study. While a low body weight does not necessarily indicate poor body condition, body weight in relation to expected weight, perhaps determined by age, breed or sex, may serve as indirect measures of poor body condition. In addition, Losada-Espinosa et al. [4] carried out a review of the validity and feasibility of a number of abattoir-based cattle welfare indicators and deemed assessment of body condition to be highly feasible. Therefore, it may be possible to systematically record body condition at ante-mortem inspection. However, this has yet to be trialled commercially. A more practicable option may be to use carcass conformation or fat coverage grades as an indirect measure of body condition. The association between body condition score and carcass conformation and fat coverage grades was assessed. As body condition became poorer, fat coverage and conformation grade decreased. Given the strong association between conformation grade and body condition in both beef and dairy cattle, carcass conformation may be a promising indirect indicator of body condition in cattle. In Europe, the EUROP carcass classification system is used and involves the mandatory recording of carcass conformation and fat coverage [63]. While other global regions such as the United States (US) and Australia have their own grading systems [64], the EUROP system could be used as an aid for identifying poor body condition in cattle across Europe. Associations between carcass condemnations and ante-mortem issues could not be assessed in the current study due to only two animals being condemned. However, Sánchez-Hidalgo et al. [56] examined associations between ante and post-mortem measures in cull cows and found that low body condition ante-mortem was associated with increased carcass condemnations. Together, these findings suggest that it is important from an animal welfare and productivity perspective to measure and consequently reduce, the prevalence of poor body condition in cattle.

### 4.3. Animal Characteristics and Additional Variables

#### 4.3.1. Associations with Carcass Bruising

Age was associated with carcass bruising in beef cattle while sex and slaughter day were associated with carcass bruising in dairy cattle. Animal characteristics such as sex or age will affect how animals cope with their environment [65]. Therefore, animals kept in similar conditions can vary in their welfare outcomes. While it is recognised that age and sex play a role in bruise prevalence in cattle, the literature surrounding this is inconsistent. For example, Mpakama et al. [36] state that young and male cattle are at greatest risk of bruising while Strappini et al. [35] have found a higher bruise prevalence in older and female cattle. Weeks et al. [66] state that bulls have the least bruising, followed by heifers, steers and cull cows, suggesting that castration status may play a role. In the current study, a distinction was not made between castrated and non-castrated animals when analysing the effect of sex. However, it can be seen in Figure 1 that, numerically, bruise frequency for bulls, steers, cows and heifers corresponds to that suggested by Weeks et al. [66]. Mendonça et al. [67] examined carcass bruising in beef cattle and found that sex was the biggest factor explaining bruise frequency with more bruising seen in females. Mendonça et al. [67] suggest that a higher reactivity in females and differences in anatomical structure between males and females may explain differences in bruise frequency. However, evidence suggests that bulls perform more aggressive and escape-related behaviour at slaughter [40,42]. This could suggest that differences in anatomic structure such as percentage of muscle tissue plays a more important role than differences in temperament. Indeed, as can be seen in Figure 2, cows had the greatest proportion of carcasses graded as ‘poor’ for conformation compared to the other sexes and the smallest proportion of carcasses graded as ‘very good,’ suggesting that they generally had poorer muscle structure than other sexes. Furthermore, Hoffman and Lühl [68] found that animals with no fat cover and the highest level of fat cover had increased levels of bruising. It is possible that very young animals with no fat coverage or older cull cows with high fat coverage may be more susceptible to bruising. Indeed, it can be seen in Figure 3 that cows had the greatest proportion of carcasses graded as ‘low fat cover’ compared to the other sexes and were also the only group to have carcasses graded as ‘very high fat cover.’ In addition, during transportation, cattle are often grouped by age, size and breed [36]. Consequently, cattle of the same age or sex may be transported, unloaded and housed together prior to slaughter, exposing them to similar conditions. Similarly, the effect of slaughter day may be due to conditions that vary from day to day such as staffing. For example, lorry drivers vary in their driving styles. Poor driving habits such as harsh breaking can cause cattle to lose their balance, increasing bruising incidence [69]. There can also be large variation in how stockpersons handle cattle including in the extent to which they hit, prod and beat them [40]. In addition, different farms will present animals on different days and these farms could vary in animal handling practices and housing conditions.

#### 4.3.2. Associations with Hot Carcass Weight

Sex and body condition were associated with hot carcass weight in beef cattle while age, body condition, slaughter day and number of moves were associated with carcass bruising in dairy cattle. Body weight is affected by sex [4] with females typically lighter than males. Furthermore, while cattle may be approaching their final skeletal height by 12 months, they continue to grow to between 6 and 8 years of age [70]. Therefore, it is logical that younger animals will have a lower carcass weight than older animals. Cattle used in the current study ranged from 1 year old to over 18 years old. Therefore, a number of animals would still have been growing at the time of slaughter. The effect of slaughter day on hot carcass weight could have been due to several factors similar to those outlined earlier including how the animals are grouped for transportation and differences in how staff members handle animals [36,40,71]. Number of moves was associated with decreased carcass weight in dairy cattle only. Recording of cattle movements has been mandatory in the United Kingdom (UK) since 1960 and most commonly refers to movements between agricultural holdings, markets and slaughterhouses [25]. Due to the unfamiliar environment and rough handling methods, cattle are particularly susceptible to stress during transportation compared to other production stages [72]. The effect of number of moves on dairy cattle carcass weight could reflect the effects of the stress of novel surroundings and mixing of unfamiliar animals associated with each transportation event. However, the date of the moves and the location type for each move were not recorded in the current study. Both factors are likely associated with the extent to which welfare issues arise, and remain evident, on the animal. More research is needed to explore associations between cattle movements and animal welfare.

### 4.4. Identifying the Source of the Damage

While issues such as poor body condition are likely to reflect long-term problems [39], others such as lameness, may reflect issues encountered at any stage of production from the farm to the abattoir. For example, Dahl-Petersen et al. [46] assessed the welfare of dairy cows before and after transportation to the abattoir and found that 15.8% of dairy cattle that were not lame on-farm had become lame during transportation. In addition, 26.6% of dairy cattle that were lame on-farm became lamer during transportation. While lameness was seen in both beef and dairy cattle in the current study, cases of more serious lameness were infrequent. Nonetheless, it would be of benefit to establish the origins of lameness. Similarly, the origin of dirty hides and skin lesions is difficult to establish. Further research is needed to compare the lifetime welfare status of cattle on-farm to that visible at ante and post-mortem inspection. One way of identifying the likely source of a particular welfare issue is to utilize routinely collected data. For example, systematic documentation of transportation lorry type, lorry driver ID and abattoir staff present per shift would allow for the identification of the likely source of welfare problems to become apparent over time [71]. Targeted action can then be taken to address welfare risks at the appropriate production stage. If bruising frequency was consistently higher when a particular team was on duty, additional training could be targeted at this group of individuals. For example, training abattoir staff to use appropriate handling techniques can reduce the incidence of bruising [5]. The utilization of pre-existing data makes welfare monitoring as efficient and inexpensive as possible [16]. The strong association between carcass conformation and body condition scores suggests that carcass grading data could be a particularly useful indirect indicator of animal welfare. Trends in this data could be examined to identity farms with a higher than normal incidence of poor body condition in their cattle.

## 5. Conclusions

Overall, the findings from the current study suggest that hot carcass weight and post-mortem bruise frequency in cattle have the potential to act as iceberg indicators of cattle welfare status ante-mortem. Data on carcass bruise characteristics could be recorded alongside currently recorded information. It may be necessary to distinguish bright red from dark red bruising if bruise age is to be estimated. However, this may not be possible in practice. Data on carcass conformation, collected under the Beef Carcase Classification Scheme, may be useful as an indirect indicator of poor body condition in both beef and dairy cattle and this warrants further investigation. In the current study, post-mortem measures varied by animal characteristics such as sex and age. These factors should be accounted for when using pre-existing data to infer the welfare status of cattle. Despite ever-evolving public health challenges, meat inspection data are often not collected with surveillance in mind. However, the current study findings suggest that pre-existing data may be useful in indicating the welfare status of cattle. Further research is needed to determine the associations between post-mortem carcass measures and the welfare status of cattle on their farm of origin.

## Figures and Tables

**Table 1:** The percentage of beef and dairy cattle respectively with each ante-mortem welfare issue.

Welfare Condition	>Severity	Breed	
		Beef (%)	Dairy (%)
Lameness	0	95.1	89
	1	4.9	8.8
	2	0	1.1
	3	0	1.1
Cleanliness	0	20.5	18.1
	1	41.0	49.5
	2	38.5	32.4
Hair loss and skin lesions *			
Body	0	63.4	74.2
	1	22.0	20.3
	2	14.6	5.5
Neck	0	89.4	87.9
	1	9.8	12.1
	2	0.8	0
Tail	0	87	88.5
	1	9.8	8.8
	2	3.3	2.7
Flank	0	36.6	39.6
	1	33.3	37.9
	2	30.1	22.2
Leg	0	26.0	22.0
	1	39.8	47.3
	2	34.1	30.8
Body condition	Underweight (score 1 or 2)	12.4	44.0
	Normal (score 3)	61.2	50.6
	Overweight (score 4 or 5)	26.4	5.4

*score 1 = hair loss, score 2 = skin lesion.

**Table 2:** Percentage of cattle with bruising of each shape and in each anatomical region.

Breed	Bruise shape	Circular	Linear	Tramline	Mottled	Irregular		
Beef		39.3	15.2	15.3	4.3	38.1		
Dairy		51.2	24.4	9.3	8.3	31.7		
	**Bruise location**	Butt	Rump	Rib	Forequarter	Pin	Hip	Back
Beef		7.4	23.8	43.6	26.4	50.8	15.6	65.6
Dairy		4.9	18.7	23.1	24.2	59.1	26.8	70.6

**Table 3:** Percentage of bruises of each colour and size.

Breed	Bruise Colour	Red	Purple	Yellow	
Beef		68.3	29.4	2.3	
Dairy		71.3	27.7	1.0	
	**Bruise size**	0–2 cm	2–8 cm	9–16 cm	Over 16 cm
Beef		74.9	6.0	13.1	6.0
Dairy		70.6	10.1	14.3	5.0

**Table 4:** Significant predictors of carcass bruising and hot carcass weight for beef and dairy cattle.

Variable	Beef		Dairy	
	CB ^1^	HCW ^2^	CB ^1^	HCW ^2^
Lameness	*p* = 0.04	*p* = 0.03		
Cleanliness	*p* = 0.02			
Skin lesions				*p* = 0.01
Hair loss				
Body condition		*p* = 0.01		*p* < 0.001
Sex		*p* < 0.001	*p* < 0.001	
Age	*p* < 0.001			*p* < 0.001
Slaughter day			*p* < 0.001	*p* < 0.001
No. of moves				*p* = 0.01

^1^ carcass bruising, ^2^ hot carcass weight.
